# University of Maryland Dental Department

**Published:** 1893-04

**Authors:** 


UNIVERSITY OF MARYLAND DENTAL DEPARTMENT.—The annual commencement was held at the Academy of Music, March 16th, 1893, at 8 P. M. The mandamus from the Board of Regents was read by the Dean, Prof. Ferdinand J. S. Gorgas, M. D., D. D. S. The address to the graduates was delivered by Rev. E. L. Watson. The class oration was delivered by C. Howard Nicholson, of Canada. The degree of Doctor of Dental Surgery was conferred on the following gentlemen by Prof. I. E. Atkinson, owing to the illness of Hon. S Teackle Wallis, LL. D., Provost of the University:

Fred. Loveland Arnold, Rhode Island; S. DeLeon Avery, South Carolina; William H. Barr, Canada; J. Edwin Boozer, South Carolina; Samuel A. Boyd, California; Samuel M. Byers, Pennsylvania; Norwood G. Carroll, North Carolina; Henry Winter Davis, Virginia; William Lee Davis, California; William W. Farmer, Virginia; Roland E. Loucke, Canada; Willie E. Minghini, W. Virginia· C. Howard Nicholson, Canada; John S. Rees, California; Thomas R. Rowe, Rhode Island; D. Fleming Sallis, Mississippi.

The new rule requiring three instead of two sessions before graduation has necessarily reduced the number of graduates this year. Last year the number was seventy three.

University prize, gold medal, for highest grade on final examination, Roland E. Loucks, of Canada; honorable mention, C. H. Nicholson, Canada.

S. S. White prize, a dental engine, H. W. Davis, Virginia; honorable mention, John S. Reese, California.

Snowden & Cowman prize, a set of Chapin A. Harris forceps, to Henry Winter Davis, of Virginia; honorable mention, John S. Reese.

The Arnold prize, set of forceps, John S. Reese; honorable mention, C. H. Nicholson.

James Hart prize, gold medal, John S. Reese; honorable mention, Roland E. Loucks.

S. S. White prize, set of Vainey's instruments, W. Lee Davis, of California, honorable mention, Samuel M. Byers, of Pennsylvania.

Prof. James H. Harris prize, gold medal, W. W. Farmer, of Virginia; honorable mention, C. H. Nicholson.

Prof. F. J. S. Gorgas prize, gold medal, N. G. Carroll, of North Carolina; honorable mention, D. E. Sallis, of Mississippi.

Dr. Frank L. Woods prize, gold medal. N. G. Carroll, of North Carolina; first honorable mention, R. E. Loucks; second honorable mention, Samuel M. Byers.

Dr. John C. Uhler prize, gold medal, Samuel M. Byers; honorable mention, John S. Reese

Dr. Isaac H. Davis prize, gold medal, Wm. H. Barr, of Canada; honorable mention, C. H. Nicholson.

Dental department prize, gold medal, W. Lee Davis; honorable mention, R. E. Loucks.

Dr. H. E. Neiman prize, a vulcanizer, W. Lee Davis; honorable mention, Samuel M. Byers.

The prizes, except the University prize, were awarded according to the judgment of twenty-five dentists, who were invited to act as judges by the dean of the school, Dr. F. J. S. Gorgas.

